# Chronic leg ulcers in a patient with Hyperoxaluria type 1: a rare and challenging diagnosis

**DOI:** 10.1093/omcr/omaf068

**Published:** 2025-06-27

**Authors:** Soukayna Kabbou, Ouiam Eljouari, Salim Gallouj

**Affiliations:** Dermatology Department, University hospital center Mohammed VI, Tangier 40000, Morocco; Dermatology Department, University hospital center Mohammed VI, Tangier 40000, Morocco; Dermatology Department, University hospital center Mohammed VI, Tangier 40000, Morocco

**Keywords:** hyperoxaluria, calciphylaxis, cutaneous oxalosis, renal disease

## Abstract

Calciphylaxis is an uncommon yet highly severe condition characterized by systemic medial calcification of arterioles, leading to ischemia and subsequent tissue necrosis. we report the case of a 29 year-old female suffering from primary hyperoxaluria type 1 and end-stage renal disease, Developed multiple painful ulcerations on her legs, initially believed to be due to cutaneous oxalosis. However, pathology findings revealed calciphylaxis. The association calciphylaxis and hyperoxaluria is rare, making this case unique. and she was treated with medical therapy, high-flow oxygen therapy and advanced wound dressings to facilitate granulation tissue formation and optimize ulcer healing, and daily hemodialysis with a low-calcium dialysate.

## Introduction

Primary hyperoxaluria (PH) is a rare autosomal recessive disorder characterized by excessive oxalate production, resulting from genetic mutations in enzymes essential for oxalate metabolism in the liver [1]. The most severe form is PH type 1 (PH-1), resulting from a lack of the liver-specific peroxisomal enzyme alanine-glyoxylate aminotransferase (AGXT) [2]. Clinical manifestations vary from nephrocalcinosis in infants to renal dysfunction and recurrent kidney stones in adults, along with Oxalosis, which refers to the accumulation of calcium oxalate crystals in multiple tissues, such as bones, blood vessels, and skin, where it can manifest in several ways, often presenting as papules, nodules, or plaques, these lesions may progress to ulceration, and further complications by increasing the risk of infections. Calciphylaxis is a skin ischemic infarction resulting from the occlusion of blood vessels in the subcutaneous tissue and dermis. This condition typically impacts patients with end-stage renal disease (ESRD) [3]. It is also seen in those with earlier phases of chronic kidney disease, as well as, in rare cases, in patients with normal kidney function. Calciphylaxis leads to intense pain and has a high susceptibility to infections, rendering calciphylaxis extremely crippling, with a yearly mortality rate of 40% to 80% [4].

## Case report

**Figure 1:**
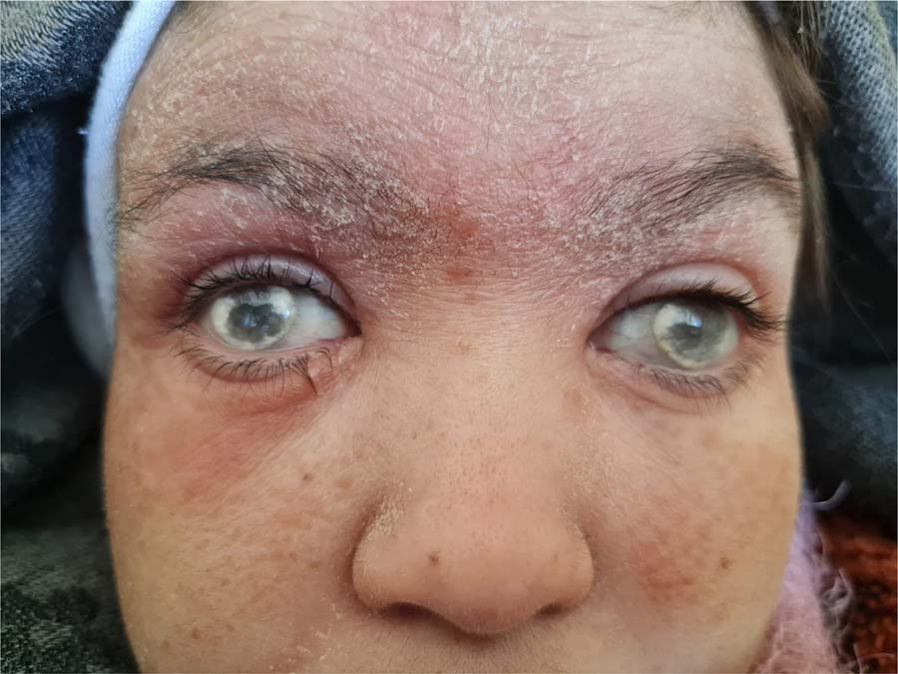
Opacification of corneas with a white-gray deposit.

**Figure 2:**
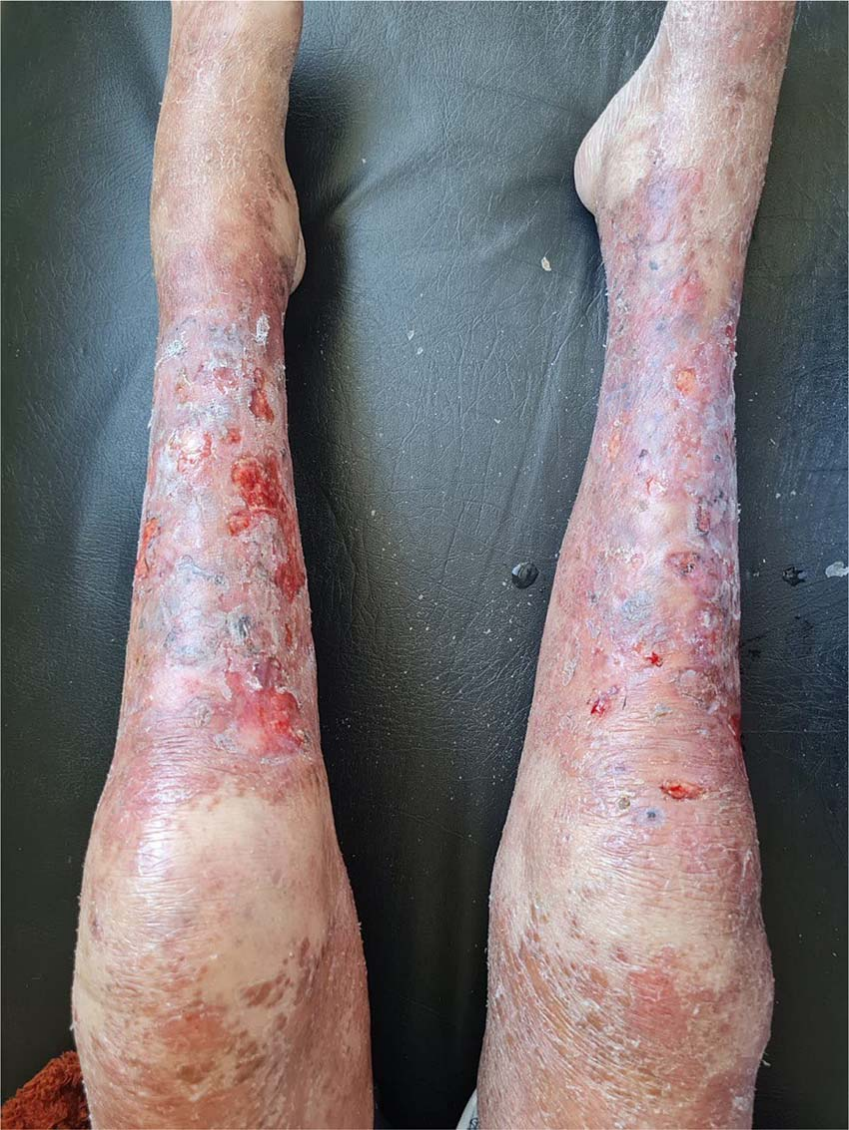
Multiple ulcerations with irregular borders, violaceous discoloration surrounding the ulcers.

**Figure 3:**
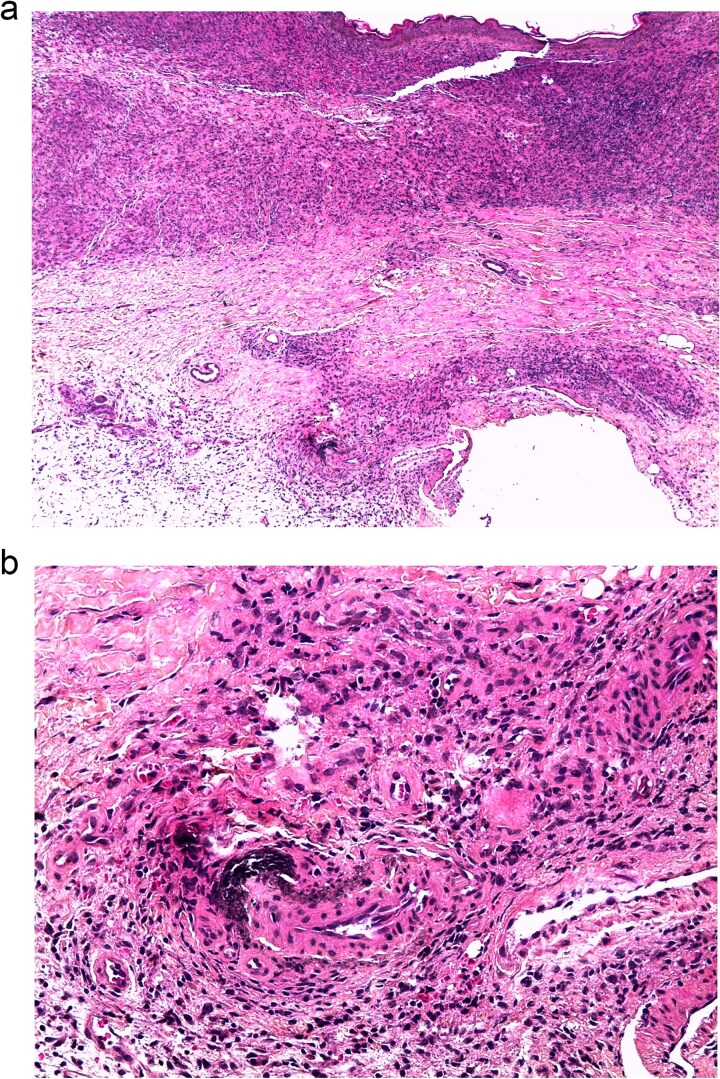
Microscopic image: A vessel with a calcified wall at the dermohypodermal junction. (b) Coloration HES G x 50. (b) Coloration HES G x 200.

A 29-year-old woman with a history of nephrocalcinosis, hyperoxaluria, and end-stage renal disease (ESRD), managed with hemodialysis three times per week.The patient developed progressive bilateral blindness due to retrocorneal oxalate deposition (Fig. 1), which became complete by the age of 6. She had a presumed diagnosis of primary hyperoxaluria type 1 (PH1). Over the past few months, she developed multiple painful ulcerations on her legs. Clinical examination revealed generalized xerosis with scaling, more noticeable on her face and limbs, consistent with chronic uremic xerosis secondary to her End-stage renal disease (ESRD), multiple ulcers with granulating surfaces, irregular borders, violaceous discoloration of the perilesional skin, and a "leather-like" appearance (Fig. 2). Based on these findings, two diagnoses were considered calciphylaxis and oxalosis. Laboratory investigations found severely elevated plasma oxalate levels (323 μmol/L pre-hemodialysis and 68.62 μmol/l post-hemodialysis) with normal levels of sodium (140 mmol/l), potassium (4.5 mmol/l), chloride (101 mmol/l), calcium (2.02 mmol/l), and phosphate (1.65 mmol/l). Genetic testing confirmed primary hyperoxaluria type 1 (PH1) with two pathogenic AGXT gene mutations: c.33dupC and Gly170Arg. A percutaneous core needle biopsy of the ulcer site revealed mild acute and chronic inflammation, fibrosis, and calcific deposits in the walls of small-caliber venular vessels at the junction between the deep dermis and superficial hypodermis (Fig. 3). Based on the clinical, laboratory, and pathology findings, the diagnosis of calciphylaxis was confirmed. The patient was admitted to the Nephrology service for medical optimization. Management included daily hemodialysis with a low-calcium dialysate, intravenous sodium thiosulfate to mitigate vascular calcification, and the use of non-calcium-containing phosphate binders to control hyperphosphatemia. Pyridoxine supplementation was also initiated to address PH1. Supportive care involved high-flow oxygen therapy and advanced wound dressings to facilitate granulation tissue formation and optimize ulcer healing.

## Discussion

Calciphylaxis, also known as calcific uremic arteriolopathy (CUA), is an uncommon yet notably severe condition characterized by systemic medial calcification of arterioles, leading to ischemia and subsequent tissue necrosis [4]. CUA is an advanced, life-threatening vascular disease that impacts small arteries measuring between 40 and 600 μm. The condition is most frequently observed in individuals with end-stage renal disease (ESRD), especially those that are dialysis-dependent, and characterized by the progressive accumulation of calcium-phosphate within arteriolar walls, leading to tissue ischemia. Cutaneous calciphylaxis is An underrecognized clinicopathologic entity, it causes painful skin lesions, the initial manifestations might consist of induration, plaques, nodules, livedo, or purpura [5]. A dark discoloration of the skin suggests a region of imminent necrosis. Often, there are netlike (reticulate) regions of erythema and livedo. Patients generally present with numerous bilateral lesions, accompanied by surrounding skin showing a leatherlike texture. The early lesions rapidly progress to stellate malodorous ulcers with black eschars. Sepsis starting from the resulting injuries is regarded as the leading cause of mortality. Calciphylaxis lesions can be classified as central, affecting the abdomen or thighs, or peripheral, affecting the extremities. In this patient, the lesions are exclusively localized in the lower extremities, consistent with the peripheral subtype. Also, calciphylaxis lesions may present as nonulcerated plaques in the early stages, progressing to ulcerated lesions in advanced stages, as observed in this case [6].

The association between calciphylaxis and hyperoxaluria is rare, making this case unique. In our patient, the lesions caused diagnostic uncertainty, because of the similarities to cutaneous oxalosis. However, This diagnostic challenge was reported in a case of a 31-year-old female with primary hyperoxaluria type 1 (PH1) and end-stage kidney disease (ESKD), who developed severe peripheral vascular disease necessitating limb amputation. Initially, her condition was presumed to be calciphylaxis, but a detailed review of the pathological specimens under polarized light revealed calcium oxalate crystal deposition within the lumen of blood vessels, leading to a diagnosis of systemic oxalosis [7]. Both cases highlight the importance of calciphylaxis and oxalosis in the differential diagnosis of vascular and cutaneous lesions in patients with hyperoxaluria and renal dysfunction, as well as the critical role of histopathology in distinguishing between these two conditions, calciphylaxis is characterized by mural calcification, microthrombi, and fibroblastic intimal proliferation, often associated with panniculitis and/or fat necrosis. In contrast, oxalosis is defined by the deposition of calcium oxalate crystals in the medial layer of arteries, leading to vascular occlusion. The management of calciphylaxis typically does not produce acceptable outcomes. Consequently, prevention is the mainstay of treatment of CUA are supportive, which involves intensive local wound management and systemic antibiotics to manage infection when necessary [8]. Frequent hemodialysis with a low calcium bath, restrict phosphate intake, utilize non-calcium phosphate binders, and employ sodium thiosulfate. Hyperbaric oxygentherapy could serve as a crucial complement to the goal of improving oxygenation in tissues [9]. In conclusion, diagnosing calciphylaxis requires a strong level of suspicion in uremic patients exhibiting distinctive lesions against a background of abnormal biochemical findings.
